# Circadian Rhythms in Visual Responsiveness in the Behaviorally Arrhythmic *Drosophila* Clock Mutant *Clk^Jrk^*

**DOI:** 10.1177/0748730417735397

**Published:** 2017-11-27

**Authors:** Olivia M. Nippe, Alex R. Wade, Christopher J. H. Elliott, Sangeeta Chawla

**Affiliations:** * Department of Biology, University of York, Heslington, York, UK; † Department of Psychology, University of York, Heslington, York, UK; 1. School of Life Sciences, University of Warwick, Coventry, UK

**Keywords:** electroretinogram, contrast sensitivity, *Clk^Jrk^*, photoreceptor, SSVEP

## Abstract

An organism’s biological day is characterized by a pattern of anticipatory physiological and behavioral changes that are governed by circadian clocks to align with the 24-h cycling environment. Here, we used flash electroretinograms (ERGs) and steady-state visually evoked potentials (SSVEPs) to examine how visual responsiveness in wild-type *Drosophila melanogaster* and the circadian clock mutant *Clk^Jrk^* varies over circadian time. We show that the ERG parameters of wild-type flies vary over the circadian day, with a higher luminance response during the subjective night. The SSVEP response that assesses contrast sensitivity also showed a time-of-day dependence, including 2 prominent peaks within a 24-h period and a maximal response at the end of the subjective day, indicating a tradeoff between luminance and contrast sensitivity. Moreover, the behaviorally arrhythmic *Clk^Jrk^* mutants maintained a circadian profile in both luminance and contrast sensitivity, but unlike the wild-types, which show bimodal profiles in their visual response, *Clk^Jrk^* flies show a weakening of the bimodal character, with visual responsiveness tending to peak once a day. We conclude that the *Clk^Jrk^* mutation mainly affects 1 of 2 functionally coupled oscillators and that the visual system is partially separated from the locomotor circadian circuits that drive bouts of morning and evening activity. As light exposure is a major mechanism for entrainment, our work suggests that a detailed temporal analysis of electrophysiological responses is warranted to better identify the time window at which circadian rhythms are most receptive to light-induced phase shifting.

## Introduction

The ability of organisms to make anticipatory changes in behavior and physiology in tune with daily environmental changes is attributed to the presence of cellular circadian clocks. The most robust and predictable environmental change that occurs during daily cycles is the intensity of light, which can change over 8 orders of magnitude within a 24-h period. The visual system undergoes structural and physiological alterations to maintain optimal visual acuity over this large luminance range such that daily and circadian rhythms in visual sensitivity have been reported across species from mammals to invertebrates. In humans, time-of-day variations have been reported in visual psychomotor responses (Stolz et al., 1988) and in evoked electrophysiological responses of visual circuits (Hankins et al., 1988; Hankins et al., 2001; Stolz et al., 1987). Electroretinograms (ERGs), extracellular neuronal recordings at the eye that reflect the field potential changes in response to a flash of light, have been used to assess rhythms in the electrical activity of neurons in the mammalian visual system. An analysis of the ERG components indicates that both the excitation of photoreceptors and postsynaptic responses of second-order neurons display a characteristic circadian profile in rodents (reviewed in Cameron et al., 2008).

The rhythms in mammalian visual sensitivity are mirrored in the genetically tractable model organism *Drosophila melanogaster*. Daily rhythms occur in ERGs (Chen et al., 1992), optomotor turning behavior (Barth et al., 2010; Mazzotta et al., 2013), along with structural alterations in the size of the photoreceptor terminals (Barth et al., 2010) and the size and morphology of the second-order lamina neurons (Pyza and Meinertzhagen, 1999; Górska-Andrzejak et al., 2005; Weber et al., 2009). Once entrained, these patterns persist in constant darkness.

Circadian rhythms in *Drosophila* visual circuits are of particular interest not only because they have to ensure adaption of the eyes to the daily changes in light but also because light is a key zeitgeber for the entrainment of the central clock neurons in *Drosophila* via visual and nonvisual input pathways (Yoshii et al., 2015). The visual inputs convey light signals to the clock neurons via the compound eye photoreceptors, via the ocelli, or via the specialized Hofbauer-Buchner eyelets (Rieger et al., 2003). Nonvisual pathways for photoreception in clock neurons rely on the blue-sensitive cryptochrome pigment (Stanewsky et al., 1998; Emery et al., 1998).

All *Drosophila* cells including the central clock neurons are equipped with a genetic time-keeping mechanism that involves rhythmic transcription of genes whose protein products feedback to inhibit their own transcription. This transcription-translation feedback loop (TTFL) is conserved in *Drosophila* and mammals (Panda et al., 2002). In *Drosophila*, *period* (*per*) and *timeless* (*tim*) are the 2 clock genes that autoregulate their transcription by inhibiting transcriptional activity of a heterodimer composed of CLOCK (CLK) and CYCLE (CYC). A second cellular timing apparatus, a metabolic oscillator, generates rhythms in the oxidation state of peroxiredoxins (Edgar et al., 2012; Rey et al., 2016), is conserved across species, and can function in the absence of the TTFL (O’Neill et al., 2011; O’Neill and Reddy, 2011). Circadian rhythms in the morphological changes of lamina neurons are abolished in mutant flies that are null for the *per* gene (*per^01^*; Weber et al., 2009; Barth et al., 2010) as are the circadian changes in optomotor responses (Barth et al., 2010). In contrast, visual sensitivity rhythms are unaffected in *per^01^* mutants (Chen et al., 1992). Thus, it is unclear whether visual rhythms require a functional TTFL and/or metabolic oscillator.

Here we examined visual sensitivity in the *Clk* gene mutant (*Clk^Jrk^*), which is behaviorally arrhythmic (Allada et al., 1998), to determine whether the TTFL is dispensable for oscillations in visual function. To test this, we deployed the conventional flash electroretinogram (fERG). ERGs performed on a dark background measure the response to a light flash while the visual system is in a dark-adapted state. The electrical response from the eye therefore gives a measure of the luminance response of the eye. The contrast of a flash of light delivered in the ERG assay is poorly defined: if it is expressed as a fraction of the mean background, then it is many hundreds or even thousands of a percentage change. We therefore deployed a highly sensitive steady-state visually evoked potential (SSVEP) assay (Afsari et al., 2014), which measures the response to a flickering light. This assay measures responses to modulations around a mean luminance, a situation that is representative of natural scenes (Laughlin, 1981). By using different frequencies and light levels, the SSVEP can sweep out the entire contrast response profile of the visual system (Norcia et al., 2015). Because the SSVEP measurements are based on a much larger number of events than a flash ERG and because the precise modulation frequency of the SSVEP inputs allow us to ignore most broadband noise, the signal-to-noise ratio of the SSVEP technique is much higher than that found in single-trial ERG experiments. These properties make the SSVEP assay sensitive and a reliable indicator of physiologically relevant visual function while also allowing comparisons with human contrast sensitivity. Finally, a systems identification approach to the SSVEP data distinguishes the response of 3 key components of the fly visual system: photoreceptors, second-order lamina neurons, and third-order medulla neurons.

## Materials and Methods

### Fly Stocks

Vials of *Drosophila melanogaster* were kept on a yeast-sucrose-agar food medium (Carpenter, 1950). The *Clk^Jrk^ st^1^* mutant (Bloomington Stock 24515, hereafter *Clk^Jrk^*) was compared with its background *st^1^* (Stock 605) and with the white-eyed standard *w^1118^* (*w*¯; University of York stock). All vials were kept at 25 °C with a 12 h:12 h light:dark schedule. Adult flies were collected within ~18 h of eclosion. They were photoentrained in 12 h:12 h lights-on:lights-off (LD) cycles for ~5/6 days in a constant temperature room (25 °C), before being transferred to constant darkness (DD) and constant temperature (again 25 °C).

### Electroretinograms

**Figure 1.**
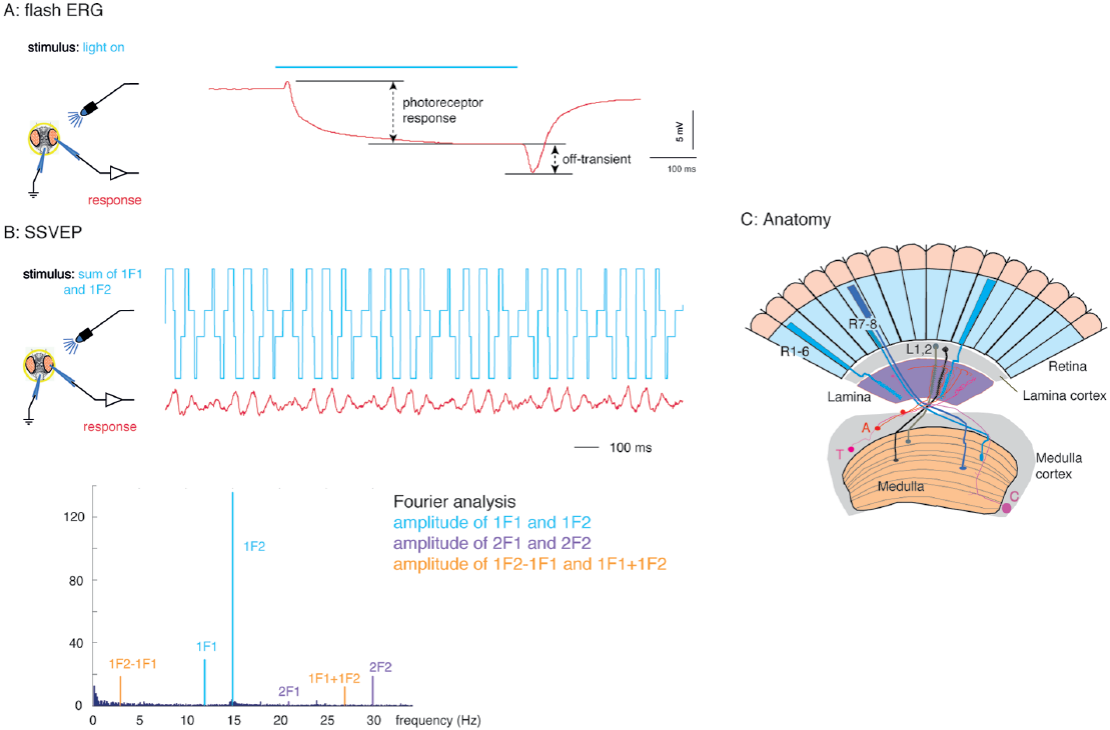
Experimental setup for recording the visual neurophysiological response of *Drosophila*. Flies were restrained with nail polish in a pipette tip. A recording electrode placed on one eye and a second, indifferent earthed electrode placed in the mouthparts. (A) For the flash electroretinogram, which measures the luminance response, a pulse of constant blue light from a light-emitting diode (750 ms) was given, and the recorded receptor potentials and off-transients were measured as indicated by the dashed lines. (B) For the steady-state visual evoked potential stimulus, which measures the contrast sensitivity, a flickering blue light was applied. The intensity of the light is the sum of 2 square waves: one at 12 Hz and the other at 15 Hz. In each trial, the amplitude of each component wave was determined randomly. The amplitude of each frequency in the response was determined using the Fourier transform, giving rise to harmonics (1F1, 2F1 . . .) and intermodulation terms (1F1+1F2, 1F2-1F1, 2F1+2F2, . . .). These frequency components are related to the anatomy of the fly eye (C), with the 1F1 component arising from the photoreceptors, the 2F1 from the lamina, and second-order neurons and the intermodulation terms (2F1+2F2) from the medulla.

Flash ERGs and SSVEP were made as described by Hindle et al. (2013) and Belušič (2011), and Afsari et al. (2014), respectively, with additional steps to avoid disrupting the circadian rhythm. Flies were trapped in a shortened Gilson pipette tip with the head and fore legs exposed (Fig. 1A,B) and secured with a small amount of nail polish (Creative Nail Design). Each fly was allowed to recover in the dark for a period of ~20 min. Recordings were made with glass electrodes filled with *Drosophila* saline, one resting on the eye, the other placed in the mouthparts. In the case of flies that were currently experiencing subjective night or were under constant conditions, this preparation process was performed under a red light to minimize interference with the flies’ current light cycle (Chiu et al., 2010). fERGs were recorded using Dasylab (Measurement Computing Corporation, 2012), analysis performed using custom Dasyview software (http://biolpc22.york.ac.uk/dasyview), and the peak-to-peak (max to min) height, receptor potential, and off-transients measured. SSVEP stimulation recording and analysis was achieved with Matlab. We presented 18 random contrast stimuli to each fly, with the light being flickered about the mean light intensity at 12 Hz (hereafter 1F1). This generates responses that the fast Fourier transform analysis identifies at the input frequency (1F1) and at twice the input frequency (2F1). Genetic dissection shows that these 2 components are due to the photoreceptors and lamina neurons, respectively (Afsari et al., 2014). In some stimuli, the 1F1 input was combined with a second input at 15 Hz (1F2, see Fig. 1B). This results in a combined “beating” pattern in which the amplitude of the response changes at the sums and differences of the input frequencies (1F2–1F1, 1F1+1F2, and 2F1+2F2). This “intermodulation” is the result of the activity of the medulla neurons, and like Afsari et al. (2014), we chose to report the 2F1+2F2 term, which arises in the medulla (see Suppl. Fig. S1). To remove any effects due to adaptation to the flickering light, only the last 9 responses were analyzed.

Circadian periodicity in the dark was estimated by fitting the equation

SS=C+α(sin(Ωt))+β(cos(Ωt)),

where SS is the response at time *t*, C is the overall mean, α and β are amplitudes, and Ω is the period. This equation has 1 nonlinear unknown, Ω, and will have a number of good fits, with minimal residuals. We systematically supplied values of Ω from 0.4 to 1.6 days and, for each Ω, determined the best linear fit of C, α, and β using the R procedure “lm.” The residual was plotted as a function of Ω. Once the approximate best fit Ω was determined, the values of C, α, and β were determined using the R “nls” nonlinear fit procedure. All data acquisition and analysis code is available at https://github.com/wadelab/flyCode, using the “Circadian” code set.

### Locomotor Activity Rhythms

The *Drosophila* activity monitor system (Trikinetics Inc., Waltham, MA, USA) was used to record locomotor activity as described previously (Fogg et al., 2014). Male flies were collected within ~18 h of eclosion, kept in a light- and temperature-controlled incubator (25 °C), and were photoentrained to 12 h light:12 h lights dark (LD) cycles for 3 days, and then monitored in constant darkness (DD) for a further 9 days. Locomotor activity was recorded in 2-min bins. Actograms and a Lomb-Scargle periodograms for each individual fly were generated using the ActogramJ plugin for ImageJ program (Schmid et al., 2011).

### Statistics

Analysis of variance was performed in R, using the Tukey post hoc test where required.

## Results

**Figure 2.**
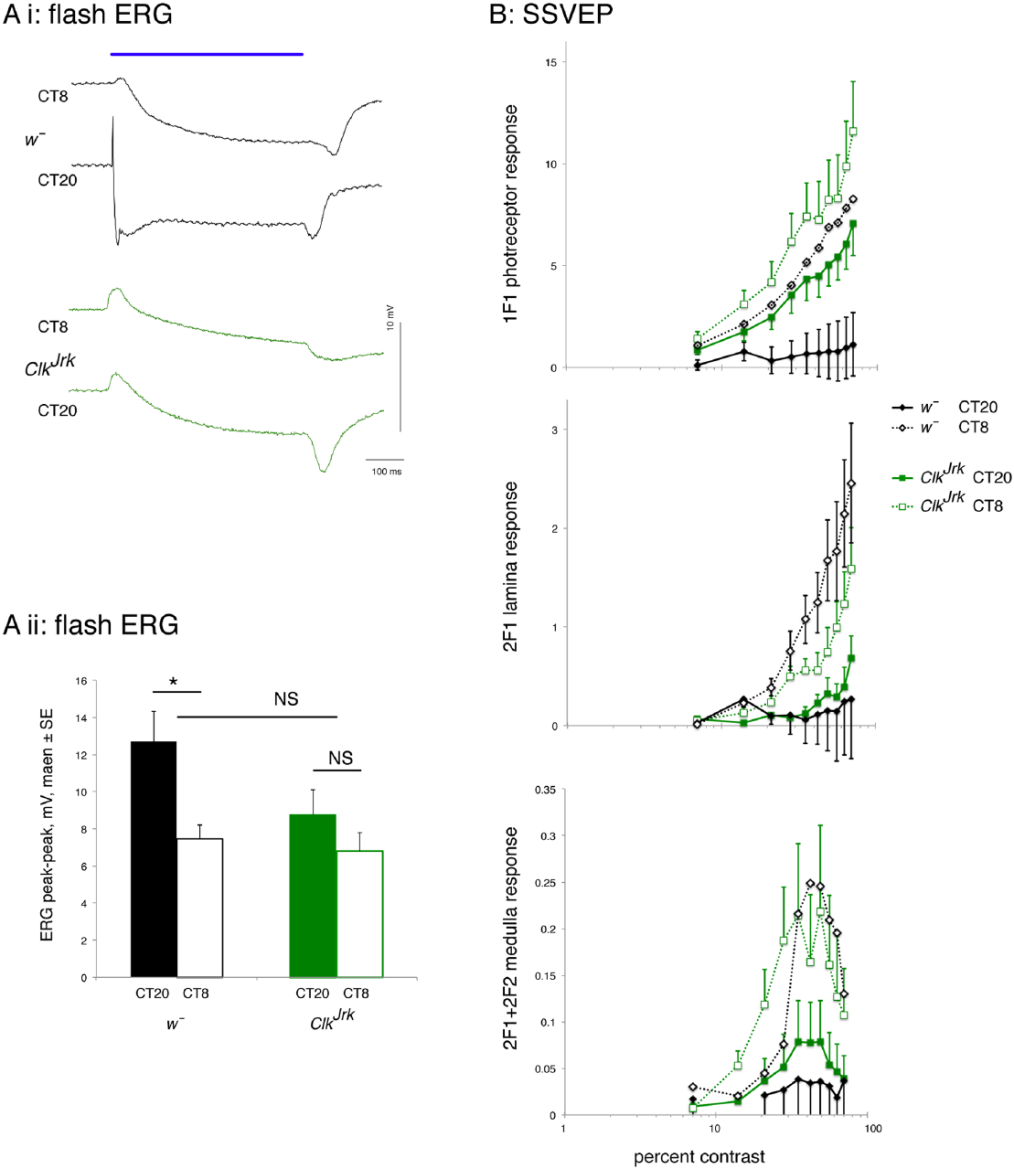
Wild-type (*w*¯) and *Clk^Jrk^* flies show different visual responses at CT8 and CT20 in DD1. (A) Qualitative (i) and quantitative (ii) differences in the flash electroretinogram (ERG) response at CT8 and CT20. Bar chart plot of the ERG peak-peak amplitude shows significant difference in the *w*¯ response between CT20 and CT8. Tukey post hoc tests showing no overall difference between *w*¯ and *Clk^Jrk^* (*p* = 0.059); a difference in the ERG of *w*¯ between CT20 and CT8 (*p* = 0.33), but no difference for *Clk^Jrk^* between these time points (*p* = 0.71). *N* = 45, at least 10 in each sample. (B) Steady-state visually evoked potential (SSVEP) contrast response functions for the photoreceptor, lamina neurons, and medulla neurons rise more steeply at CT8 than at CT20, indicating a stronger visual response to flickering light. The overall multivariate analysis of variance indicates differences in genotype (*p* < 10^–6^), time point (*p* = 0.0002155), and the genotype × time point interaction (*p* = 0.0126175). The subsequent analysis of variance indicates differences in time point for each component of the SSVEP response (photoreceptors, lamina neurons, and medulla neurons; see Suppl. Table S1). Only the photoreceptors show a difference due to genotype, while the lamina neurons show a genotype × time point interaction. Data from the same 45 flies in A. Exact genotypes: *w*¯ = *w^1118^*; *Clk^Jrk^* = *Clk^Jrk^*,*st^1^*.

We first compared the fly visual response at the end of subjective day (CT8) with that at the end of subjective night (CT20), as at these times ERG sensitivities have been previously reported to differ considerably (Chen et al., 1992). We entrained flies for 6 days and then moved them into darkness for 24 h (DD1). We first tested white-eyed flies (*w*¯) since they give a larger fERG response than red-eyed flies and observed differences in their ERGs at the 2 time points. The ERG traces of wild-type *w*¯ flies show marked differences at CT20 and CT8 (Fig. 2Ai) in both the size of the receptor potential and the amplitude of the off-transient. In contrast, the ERG traces of the scarlet-eyed *Clk^Jrk^* flies differ less in their waveforms between the 2 time points. Quantitative analysis of the ERG peak-to-peak amplitude shows that wild-type flies have on average a larger response at CT20 than CT8, whereas the *Clk^Jrk^* mutants respond similarly at CT20 and CT8. This might suggest a loss of rhythmicity in visual responses in the mutants. To investigate this further, we also compared the genotypes in the SSVEP assay. Figure 2B shows that in the SSVEP assay, the visual response of both wild-type flies and *Clk^Jrk^* mutants has a higher amplitude at CT8 than CT20, suggesting that contrast sensitivity is higher at the end of the subjective day than at the end of the subjective night. This is true for all 3 parameters measured (1F1, 2F1, and 2F1+2F2), showing that there is increased response to changes in contrast by the photoreceptors, lamina neurons, and medulla neurons at the end of subjective day.

**Figure 3.**
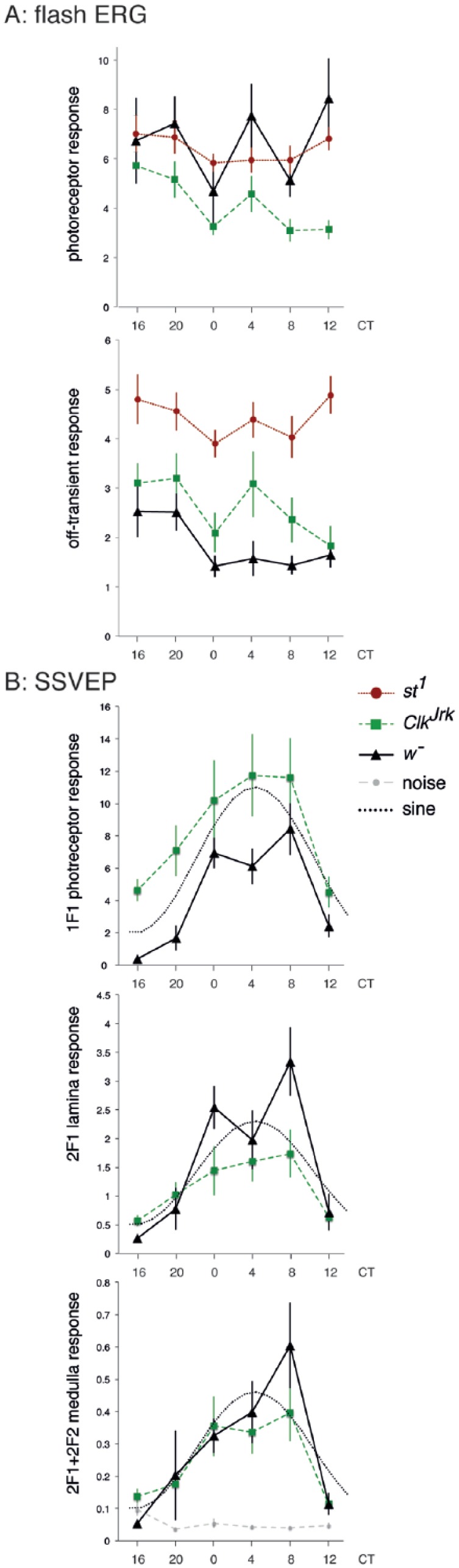
Circadian visual profile of wild-type (*w*¯, *st^1^*) and *Clk^Jrk^* flies on DD1. (A) Flash electroretinograms show peak sensitivity in the subjective night (CT16-20) and minima at CT0 and CT8-12. For both photoreceptor response and off-transient, the 2-way analysis of variance (ANOVA) shows significant effects of time of day and genotype (photoreceptor: F_5, 190df_ = 2.8, *p* = 0.019, and F_1, 190df_ = 10.5, *p* < 10^–4^ respectively; off-transient: F_5, 190df_ = 2.4, *p* = 0.035, and F_1, 190df_ = 38.4, *p* < 10^–14^, respectively), but no interaction. *N* = 207, at least 6 in each sample. (B) Steady-state visually evoked potential (SSVEP) analysis shows peak sensitivity in the subjective day for the photoreceptors, lamina neurons, and medulla neurons. The photoreceptor response is bigger for the *Clk^Jrk^* flies than the *w*¯ at all time points. The ANOVA shows significance for genotype and time but not for their interaction (genotype: F_1,131df_ = 22, *p* < 10^–5^; time: F_5,131df_ = 9.8, *p* < 10^–7^). For the neuronal responses (lamina or medulla neurons), there is no difference between the *Clk^Jrk^* and *w*¯ flies. The sensitivity of the SSVEP assay is indicated in the 2F1+2F2 (medulla neuron) trace, where the response is ~10× the noise level. The dotted line (sine) indicates a waveform with the maximum in the subjective night and minimum in the subjective day. Data from the same 135 *Clk^Jrk^* and *w*¯ flies in A, using the maximum response for each fly. Exact genotypes: *w*¯ = *w^1118^*; *Clk^Jrk^* = *Clk^Jrk^*, *st^1^*.

Given the apparent loss of rhythmicity of *Clk^Jrk^* mutants in fERGs but not in the SSVEP assay, we extended the data set and sampled flies from free-running constant darkness conditions (DD1) every 4 h (Fig. 3). We also included the wild-type strain *st^1^* here to rule out genetic background as a cause for the different response of the *Clk^Jrk^* mutants in fERGs and also analyzed the photoreceptor potential and off-transients separately. Figure 3A shows that in the fERG responses, the temporal profiles of the 3 genotypes are for the most part similar but diverge considerably at CT12. At CT12, the receptor potential of the wild-type strains (*w¯* and *st^1^*) is maximum, while for the *Clk^Jrk^* mutants, the photoreceptor response at CT12 is at its minimum. Overall, the fERG data suggest that all genotypes have a higher luminance response in the subjective night.

In the extended SSVEP assay (Fig. 3B), both genotypes show a circadian pattern, but the response is dominated by a peak in the second half of the subjective day (CT4-CT8). The photoreceptor response is stronger in the *w*¯ than in the *Clk^Jrk^* mutants, but the neural signaling components (lamina neurons and medulla neurons) are not separated by genotype. At CT4, there is a dip in the *w*¯ photoreceptor and lamina neuron SSVEP response, mirroring the photoreceptor response peak in the fERG, but this is not seen in the *Clk^Jrk^* data.

**Figure 4.**
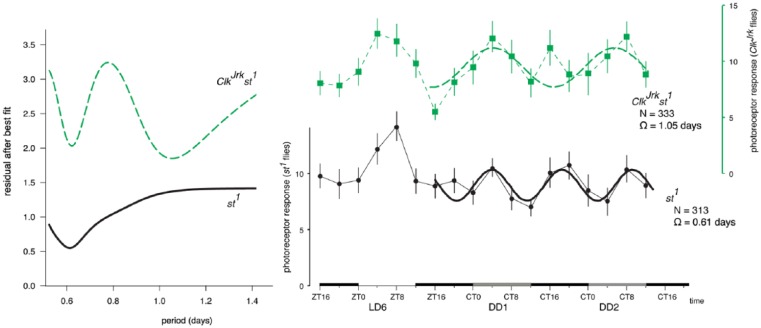
Calculating the best fit of a sine wave to the photoreceptor component of the steady-state visually evoked potential data shows the *Clk^Jrk^* flies maintain a DD rhythm with circadian periodicity, but the *st^1^* flies have a rhythm with a periodicity of ~2 cycles/day. (A) Fitting successive values of Ω, the period, shows a good fit at ~14 h for both genotypes. However, the *Clk^Jrk^* have a better fit with a period of ~1.05 days. (B) Plotting the best-fit lines shows that the *Clk^Jrk^* data are well explained by an equation with period of 25.2 ± 3.1 h, whereas the *st^1^* period is 14.6 ± 0.6 h.

To confirm our *Clk^Jrk^* data, we next examined the periodicity in detail over LD6, DD1, and DD2. We compared the *Clk^Jrk^* flies with a scarlet mutation (*st^1^*), as the *Clk^Jrk^* mutation is in the *st^1^* background. For both genotypes, the variation in 1F1 response is larger in LD6 than in DD. We fitted a periodic cycle to the DD data, determined the residuals (Fig. 4A), and found both genotypes showed a minimum in the residual at ~14 h. The *Clk^Jrk^* (but not the *st^1^*) showed a better fit for a period of 25 h. Plotting the curves shows a good fit between the data and the calculated lines (Fig. 4B), confirming that the visual sensitivity of *st^1^* flies has peaks approximately twice a day, whereas the *Clk^Jrk^* flies have a “circadian” rhythm. The peak of the *Clk^Jrk^* fitted curve is at CT4, while the peak on the last LD day is at ZT4, suggesting there is no phase shift over this time span.

**Figure 5.**
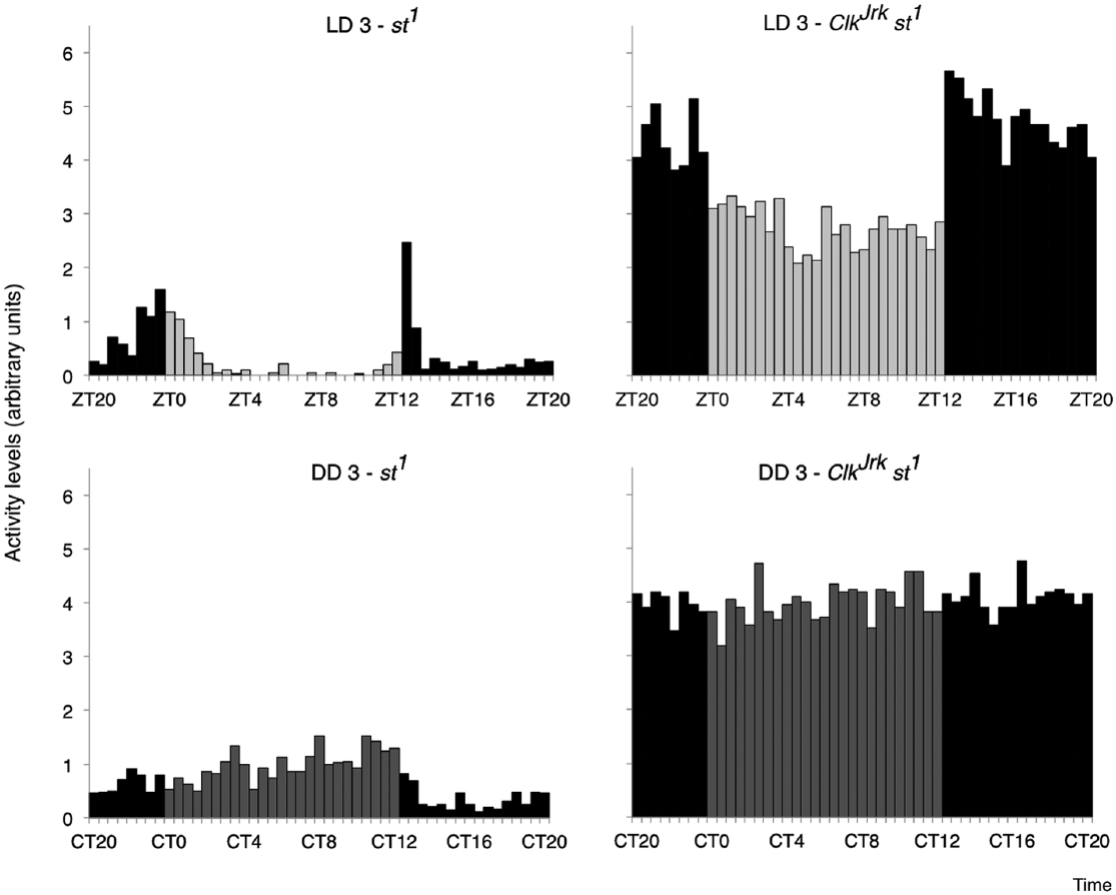
Nocturnal locomotor activity in LD for *Clk^Jrk^*,*st^1^* but not *st^1^* flies. Average daily activity profiles of *st^1^* flies (left graphs) and *Clk^Jrk^* mutants (right graphs) in 30-min bins during a 24-h period in LD cycles (data are from LD3) and during free-running constant darkness conditions (data shown from DD3). Note the elevated activity of the *Clk^Jrk^*, mutants during the dark phase of LD, and arrhythmic phenotype in DD. *N* = 54 *st^1^* and 21 *Clk^Jrk^*,*st^1^* flies.

Finally, we confirmed the locomotor phenotype of the *Clk^Jrk^* and *st^1^* flies. The scarlet-eyed control flies *st^1^* exhibit 2 clear peaks in locomotor activity levels under LD conditions, which center around light-on and off or ZT0 and ZT12 (Fig. 5). Under DD conditions, 69% of the *st^1^* flies were rhythmic (Lomb-Scargle analysis), and these had an average free-running period length of 24.4 h. The *Clk^Jrk^* mutants have a strong nocturnal rhythm under LD conditions (Kumar et al., 2012). They have relatively constant activity levels during the day, which then increased by approximately 60% 30 min after light off and remained fairly constant until ZT0. The sharp differences in activity that occur at the 2 light transitions indicate a lack of light anticipatory behavior in the *Clk^Jrk^* mutant. Under constant darkness, only 16.6% of the *Clk^Jrk^* flies were rhythmic, with mean DD period slightly lengthened at 25.2 h.

## Discussion

Here we report that in both fERG assays and SSVEP responses, visual sensitivity in *D. melanogaster* displays a notable time-of-day dependence. We have further demonstrated that the *Clk^Jrk^* mutation results in flies with a maintained circadian rhythm in visual response in constant darkness. The *Clk^Jrk^* rhythm largely recapitulates that of the wild-type *w*¯ flies both showing a higher luminance response in the subjective night and greater contrast sensitivity toward the end of the subjective day. This is surprising given that *Clk^Jrk^* flies are arrhythmic in their locomotor activity rhythms. The *Clk^Jrk^* mutants express a truncated CLK protein that retains its DNA binding and dimerization domain but lacks its C-terminal transactivation domain (Allada et al., 1998). This explains the *Clk^Jrk^* mutant’s dominant phenotype in locomotor activity rhythms as it is likely able to bind DNA and its DNA-binding partner CYC but unable to induce gene transcription.

From our initial experiments, it would seem that the genetic oscillator, the TTFL, is not required for oscillations in visual responsiveness assessed by the ERG amplitude and SSVEP assays. However, an extended time course comparing the SSVEPs of *Clk^Jrk^* with the genetically comparable *st^1^* strain revealed notable differences in their visual rhythms under DD conditions. The SSVEP photoreceptor response in *st^1^* displays an ultradian rhythm approximating to 14 h, while that of the *Clk^Jrk^* mutants oscillated with a circadian time course of 25 h. Moreover, the amplitude/duration of the *Clk^Jrk^* circadian rhythm is more robust than that of the *st^1^* flies, even though the *Clk^Jrk^* is in the *st^1^* background.

From a functional perspective, the twice-a-day contrast response in visual sensitivity in wild-type flies could map on to the need for optimal visual acuity at morning (M) and evening (E) peaks of locomotor activity in wild type flies (Helfrich-Förster, 2000).

A twice-a-day increase in the size of the L1 and L2 lamina neurons has been seen in daily rhythms (Pyza and Meinertzhagen, 1999), which might be a potential correlate of the physiological changes reported here. Similarly, a twin peak rhythm in a synaptic protein, bruchpilot, is reported in LD cycles of wild-type flies (Górska-Andrzejak et al., 2013).

In *Clk^Jrk^* mutants, a robust circadian rhythm in contrast response is more apparent because of suppression of one of the wild-type peaks in visual sensitivity, suggesting that they might be regulated separately, similar to the morning and evening peaks in locomotor activity that are controlled by different subsets of clock neurons (Grima et al., 2004; Stoleru et al., 2004) In this context, we note that in DD, only L1 laminar neurons oscillate in size in wild-type flies, being larger in the subjective night (Pyza and Meinertzhagen, 1999). Interestingly, in assessing the contribution of different neurons to contrast Joesch et al., (2010), note that L1 neurons mediate “ON” responses and L2 “OFF” responses so that circadian changes in the “ON” response pathway might explain our observation of a stronger SSVEP lamina response at the end of the subjective day. Furthermore, in DD, the levels of bruchpilot seem to display a unimodal rhythm (Górska-Andrzejak et al., 2013), although this was measured at 9-h intervals, which might miss an intervening peak. While a differential effect of *Clk^Jrk^* on the L1 and L2 lamina neurons is one possible explanation for our results, we cannot discount effects on other neurons in the visual circuit, nor can we exclude the possibility that this is the consequence of the aberrant axonal organization of the s-LNv neurons (Park et al., 2000).

It is possible that the cyclical changes in visual sensitivity reported here are controlled by the genetic clock oscillator as circadian expression of genes involved in *Drosophila* visual processes have been reported (Claridge-Chang et al., 2001; Ceriani et al., 2002). Claridge-Chang et al. (2001) observed circadian cycling of mRNAs encoding the rhodopsins *Rh4*, *Rh5*, the *trpl* receptor involved in phototransduction, the rhodopsin chaperone *ninaA* and *Pdh*, a photoreceptor dehydrogenase that participates in chromophore recycling by retinoid isomerization (Wang et al., 2010). It is noteworthy that frequent sampling of gene expression in mammalian systems has revealed mRNAs that oscillate with periods of 10 to 14 h (Hughes et al., 2009) and mRNAs that peak twice in a 24-h period (Pembroke et al., 2015). Alternatively, the maintained visual rhythms in the *Clk^Jrk^* could be due to the metabolic oscillator, which continues to generate robust oscillations in peroxiredoxin oxidation state in *Clk^Jrk^* flies, albeit with a different phase (Edgar et al., 2012). In this regard, it is interesting to note that a hypomorph CLK mutant, *Clk^AR^*, accumulated more reactive oxygen species with age than wild-type flies (Vaccaro et al., 2017).

Our findings also have implications for entraining the circadian system as light via the compound eyes can synchronize the *Drosophila* clock (Reiger et al., 2003). We would like to suggest that rhythms in visual function reported here reveal critical time windows when the *Drosophila* clock would be more receptive to light entrainment or light-induced phase shifting.

Finally, we note from our experiments that during the daily cycle, luminance sensitivity peaks in the subjective night, while the contrast response function is stronger in the subjective day. Of note, a higher contrast sensitivity in the day has also been reported in rodents (Hwang et al., 2013). Our work suggests a tradeoff between luminance and contrast. In the dark, the gain control in the eyes is relaxed, allowing photoreceptor sensitivity to be increased. A similar tradeoff exists between visual dynamic range, which was lowest at subjective night, and the optomotor response, which was lowest in subjective day (Barth et al., 2010). Our data also show faster responses (shortened latency) in the subjective night, a phenomenon also seen in the human daily visual rhythm (Hankins et al., 2001). These similarities suggest that the mechanistic basis for circadian tuning of *Drosophila* visual function can potentially provide insights into the mammalian system.

## Supplementary Material

Supplementary material

Supplementary material

Supplementary material

Supplementary material

Supplementary material

Supplementary material
