# Supplementary figures and images for: Circadian Rhythms in Visual Responsiveness in the Behaviorally Arrhythmic Drosophila Clock Mutant ClkJrk
Source: J Biol Rhythms. 2017 Nov 27;32(6):583–92. doi: 10.1177/0748730417735397 (PMC5734378; doi:10.1177/0748730417735397)

A: flash ERG

stimulus: light on

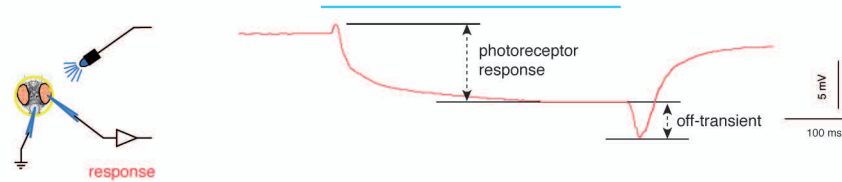

B: SSVEP

stimulus: sum of 1F1 and 1F2

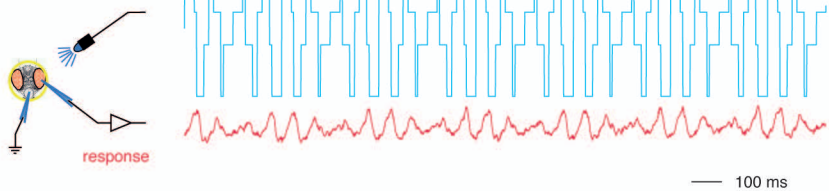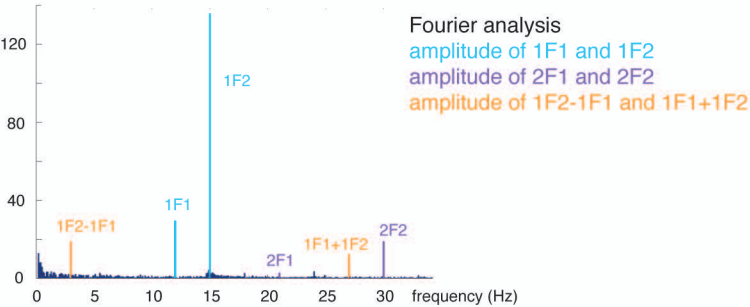

C: Anatomy

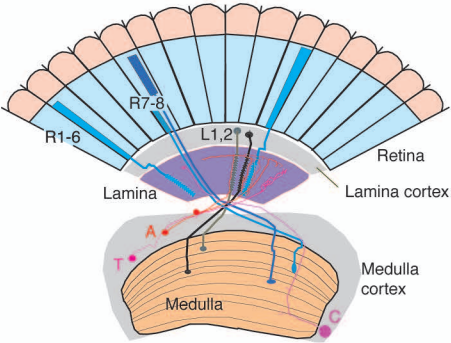

Supplement: Supplementary material [file Fig1_a.pdf]

A i: flash ERG

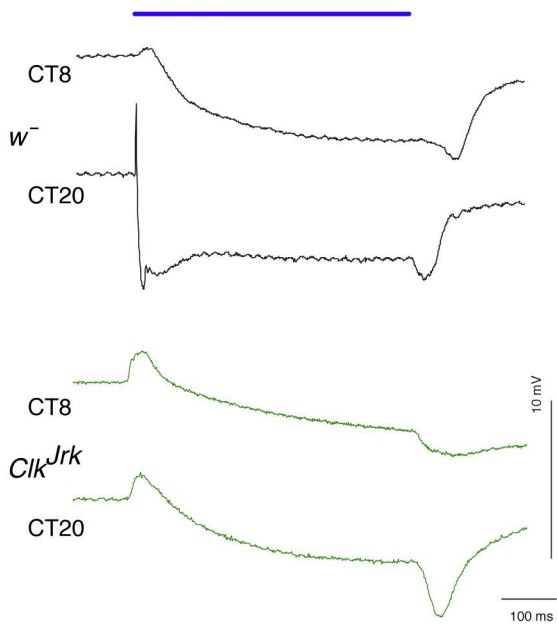

A ii: flash ERG

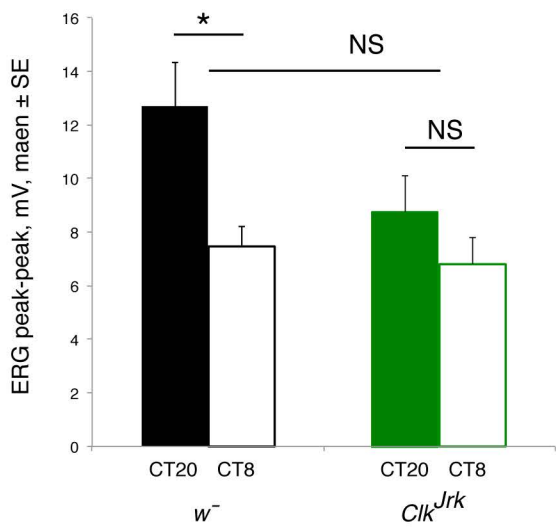

B: SSVEP

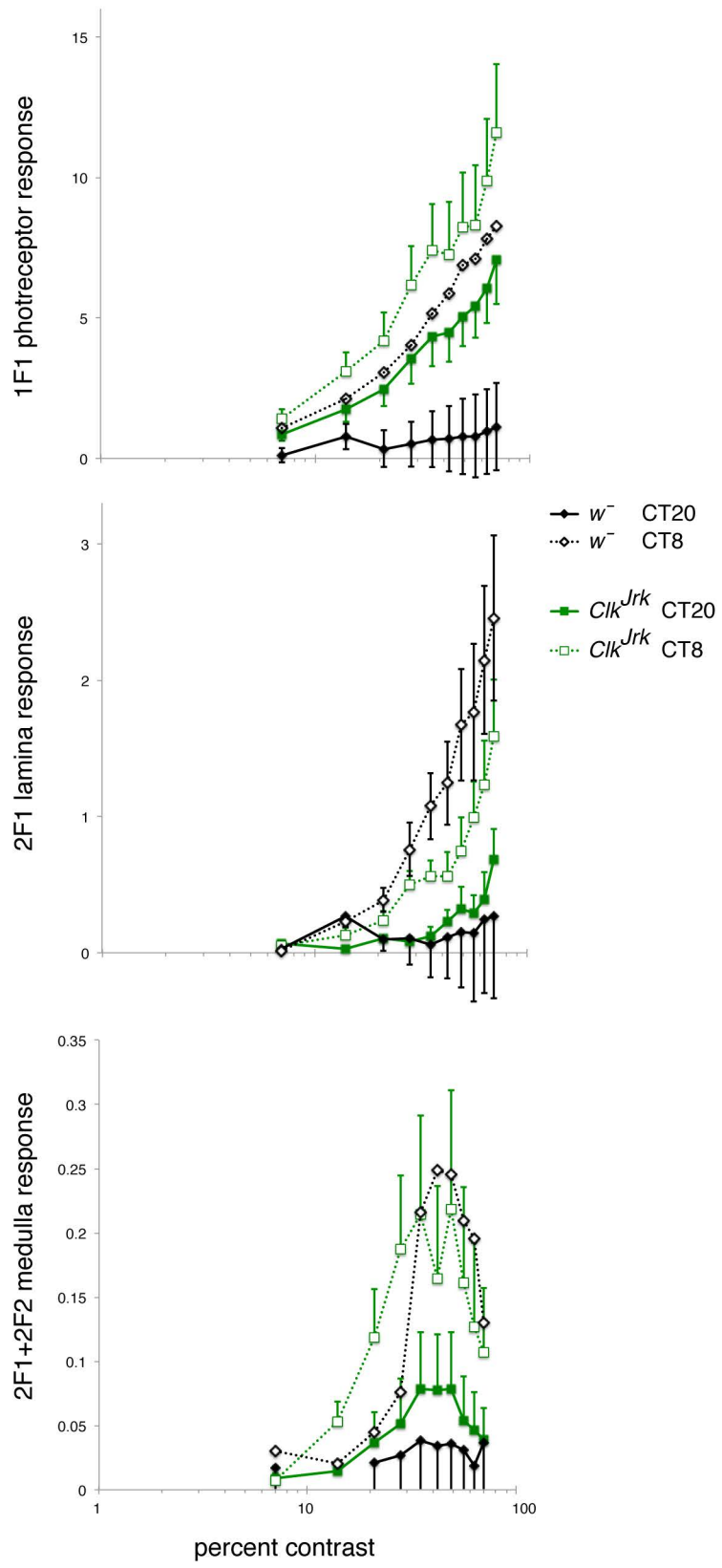

Supplement: Supplementary material [file Fig2_a.pdf]

A: flash ERG

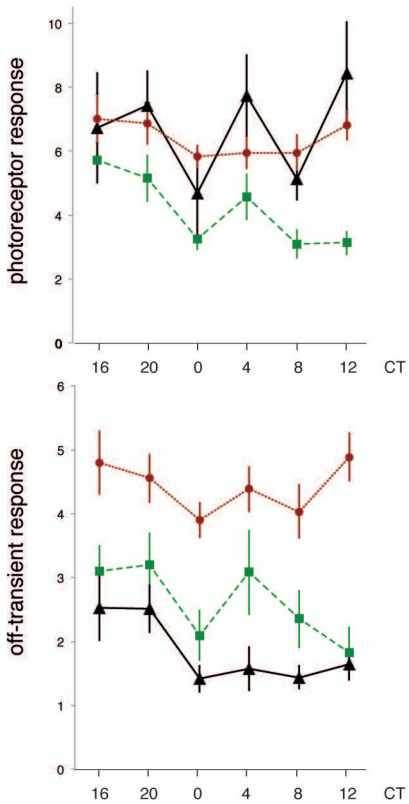

B: SSVEP

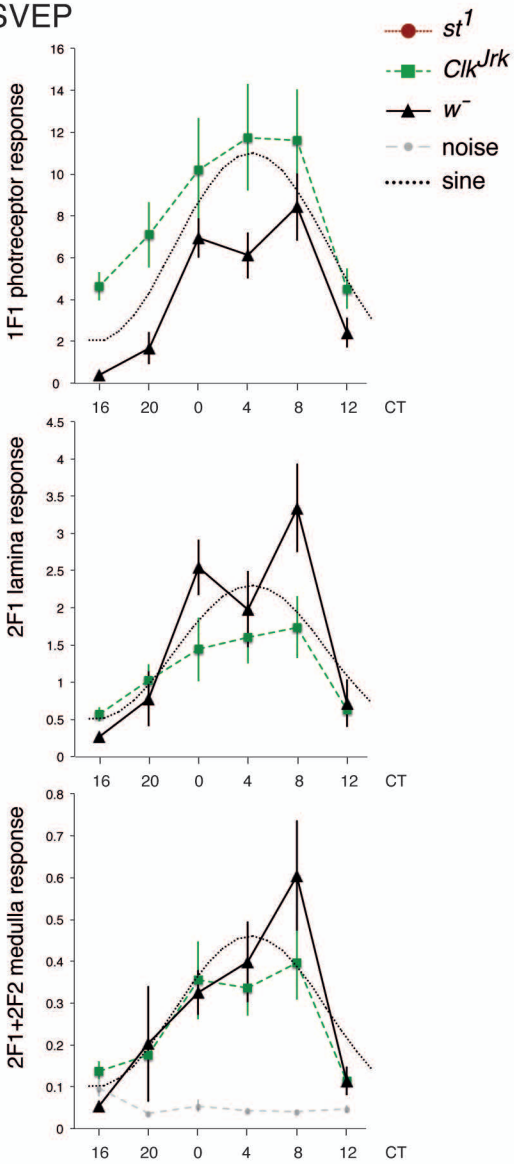

Supplement: Supplementary material [file Fig3_a.pdf]

residual after best fit

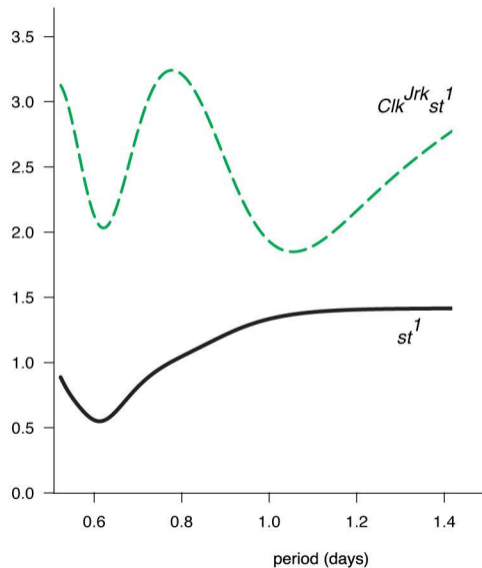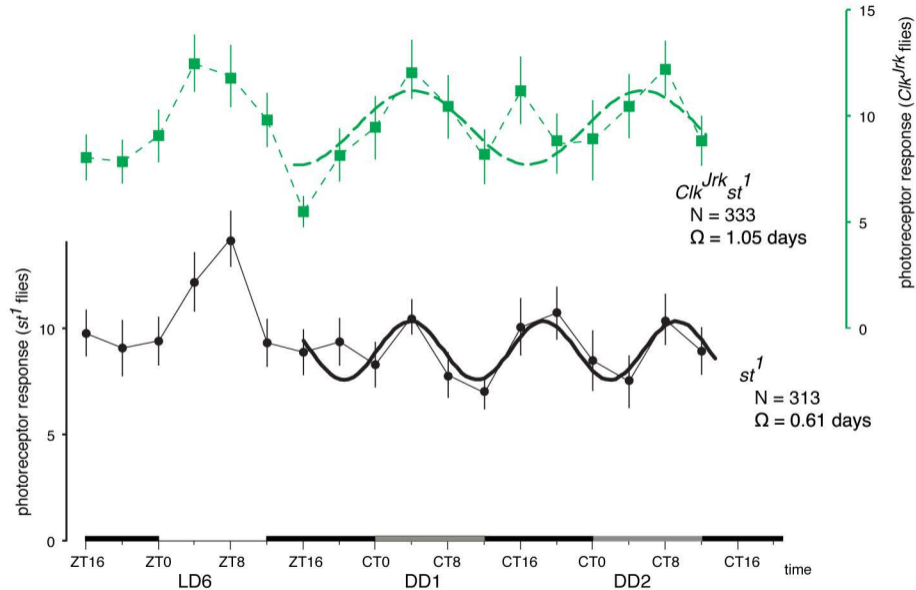

Supplement: Supplementary material [file Fig4_a.pdf]

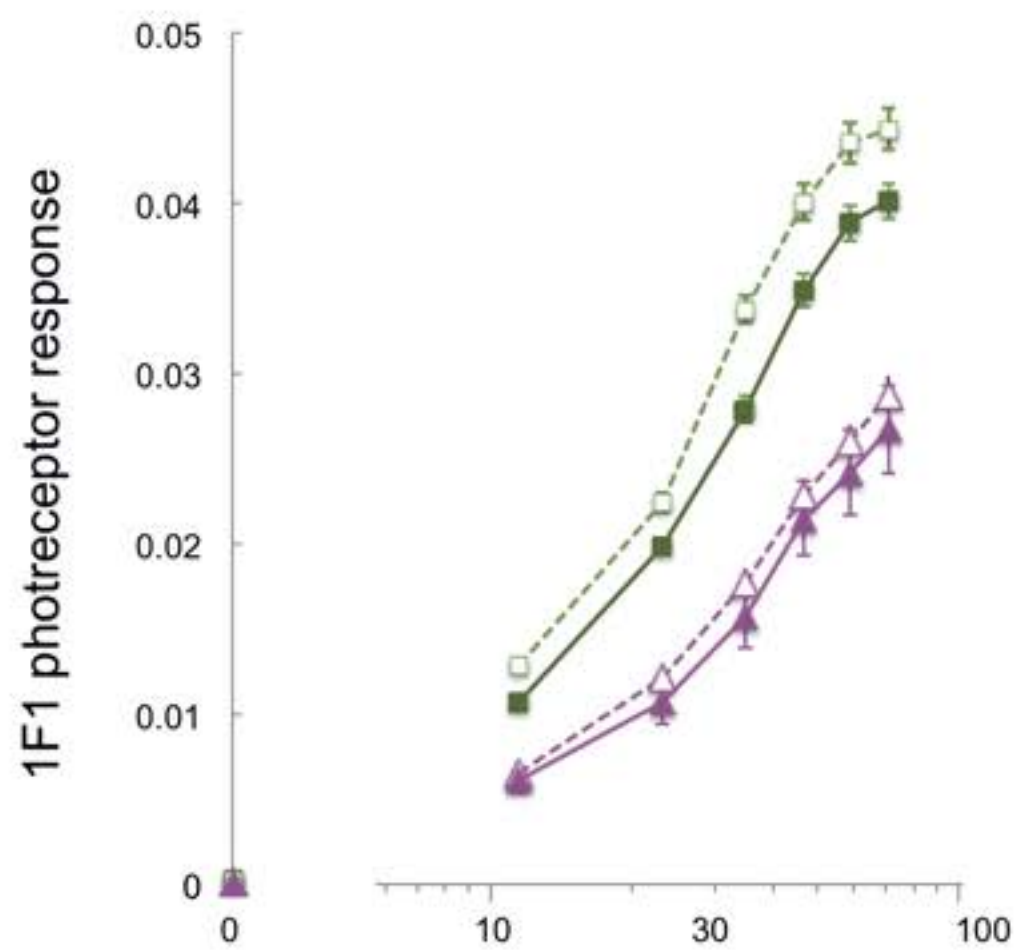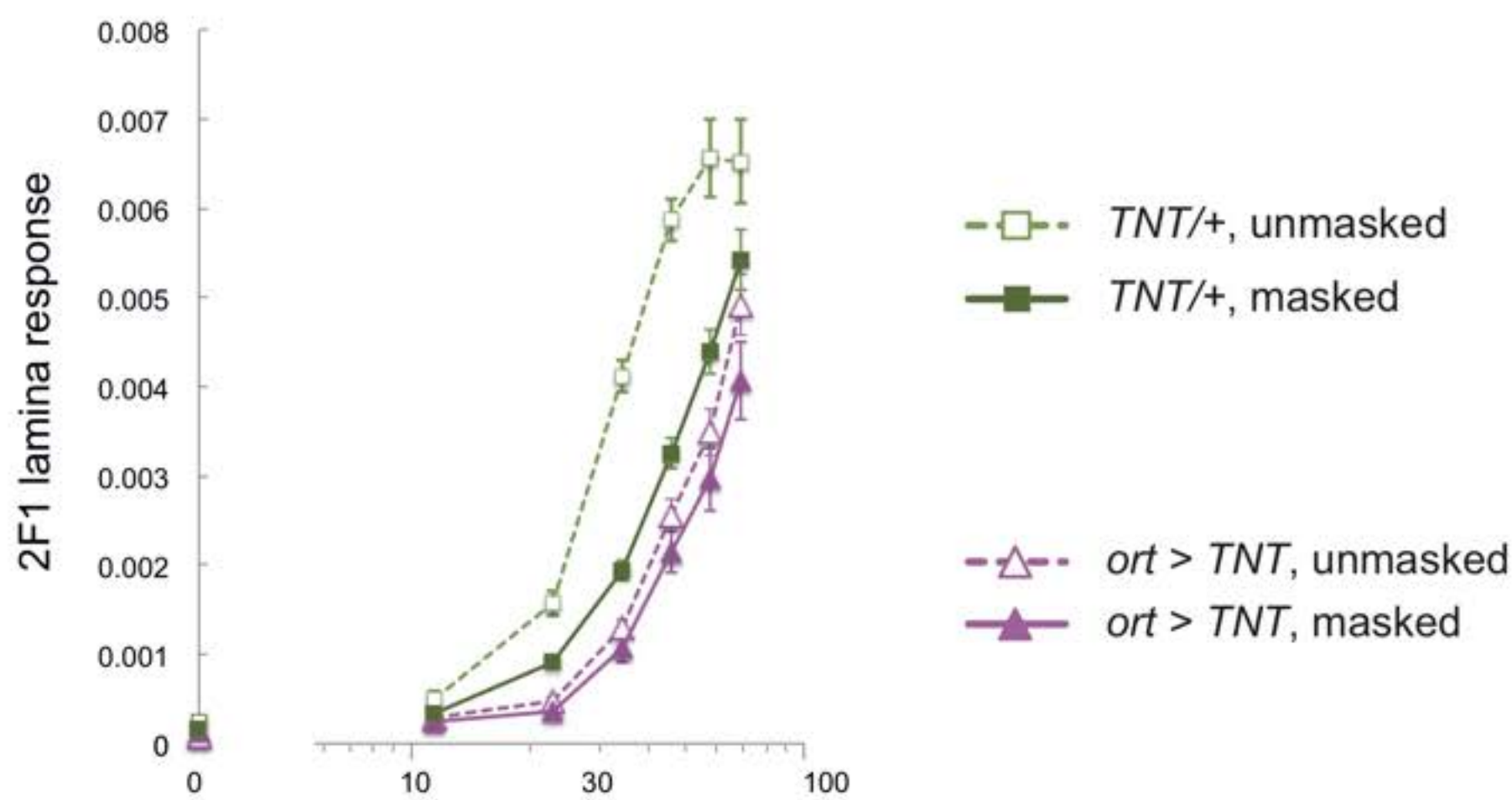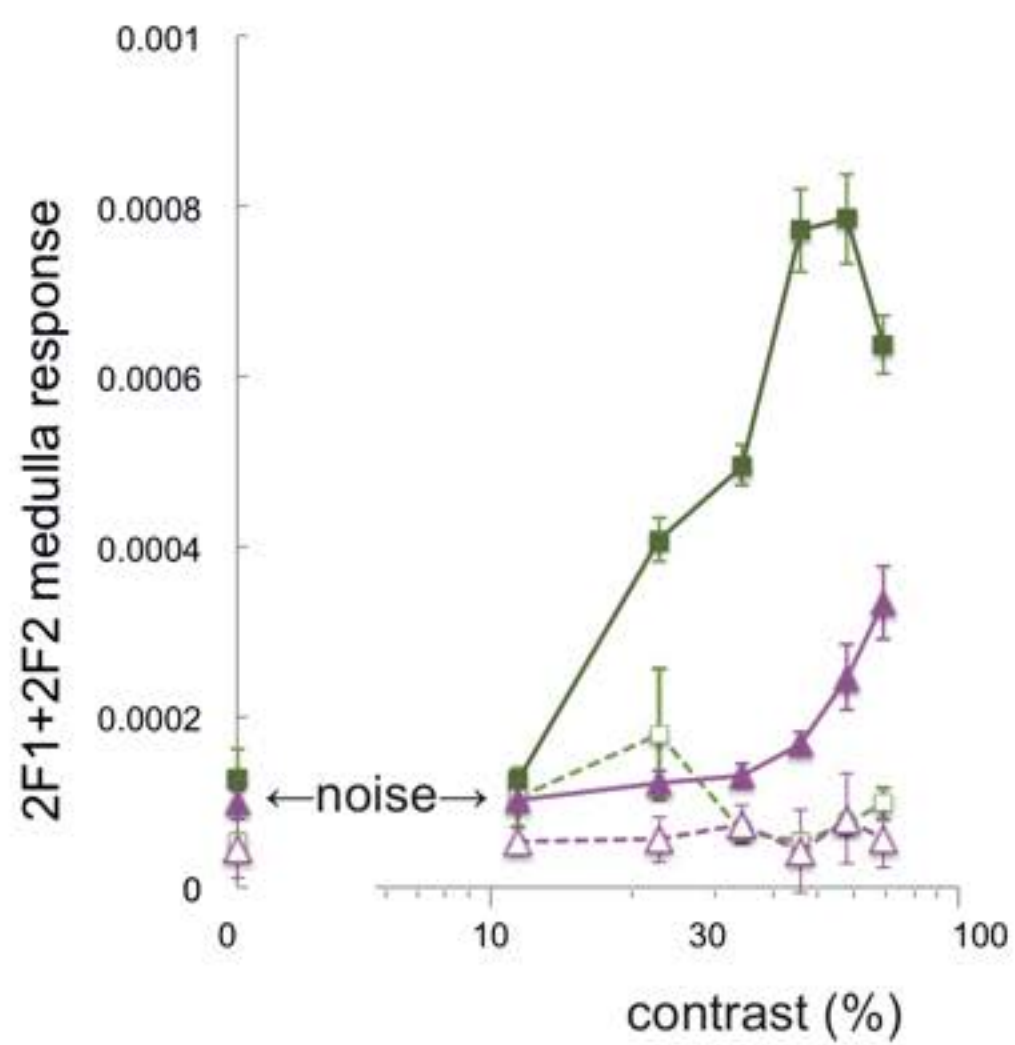

Supplement: Supplementary material [file SupplFigS1.pdf]
