# Supplementary material for: Circadian Rhythms in Visual Responsiveness in the Behaviorally Arrhythmic Drosophila Clock Mutant ClkJrk
Source: J Biol Rhythms. 2017 Nov 27;32(6):583–92. doi: 10.1177/0748730417735397 (PMC5734378; doi:10.1177/0748730417735397)
Supplement: Supplementary material [file Supplemental_material.pdf]

Nippe et al Supplementary material

Supplementary Figure S1 Legend. **Knockout of synaptic transmission from second order neurons abolishes the 2F1+2F2 medulla neuron response**. In the SSVEP (steady state visual evoked potential) analysis, we compare the response to the mix of 12 Hz (1F1) and 15 Hz (1F2) blue light stimuli with the response to a single input at 12 Hz. This scenario was designed to mimic a human watching a flickering TV monitor, with horizontal stripes at 12 Hz with or without vertical stripes at 15 Hz. Because the additional vertical stripes reduce the response to the horizontal stripes, the double stimulus is referred to as the 'masked' paradigm, the single 12 Hz stimulus (horizontal stripes) as the 'unmasked' paradigm. A Fast Fourier Transform (FFT) resolves a complex response from the fly eye into components at the frequencies supplied (1F1, 1F2), but also at other frequencies, notably a harmonic at 2F1 (24 Hz) and at the intermodulation frequency, 2F1+2F2 (54 Hz). In the flickered steady-state illumination used here, the photoreceptors follow the stimulus input and generate the 1F1 response. The lamina neurons encode transient signals, and generate a response each time the light level goes up or down, so they are principally responsible for the 2F1 response. Genetic dissection showed (Afsari et al., 2014) that flies with no histamine receptors (*ort* null) showed no 2F1 response.

Here we test the effect of knocking out synaptic transmission from the second order neurons (lamina neurons, amacrine neurons) in the fly retina. All these cells express the histamine receptor, ORT, and so we used an *ort*-GAL4 (Gengs et al., 2002) to express tetanus toxin (TNT, Sweeney et al., 1995) in these cells.

In the control fly with no transgene expressed (*TNT/+*) all three components of the masked response (1F1, 2F1 and 2F1+2F2) are clearly seen (solid green lines). The 1F1 and 2F1 components increase monotonically with the contrast, but the 2F1+2F2 component shows a winner takes all scenario, peaking at ~50 % contrast. In this respect, the fly response is similar to that of the humans watching horizontal stripes and vertical stripes, where the response of the 2F1+2F2 component shows the same peak when the contrasts are equal.

When tetanus toxin is expressed in all the second order neurons using the *ort* histamine receptor GAL4 (*ort* > *TNT*), the masked paradigm 1F1 and 2F1 responses (magenta lines) are similar to the control flies, though shifted right (or down) by the slightly darker eye colour. However, the 2F1+2F2 component is markedly different, being now much reduced and monotonically increasing with contrast.

When only 12 Hz stimulation is applied ('unmasked paradigm' with no 1F2, only 1F1), both control and *ort* knockout flies still show the 1F1 and 2F1

responses (green dashed lines), but the level of the 2F1+2F2 component is never above the noise recorded (0.0001) when no stimulation is applied.

Exact genotype: + is w^{-}. N = 10 for each cross.

[Note also that the solid and broken lines are much closer in the *ort* > *TNT* fly than in the controls, particularly in the 2F1 response, also an expected consequence of the masking paradigm, in which the third-order neurons feed back to the lamina neurons and photoreceptors.]

We conclude that the *ort* > *TNT* fly fails to show the full, normal pattern of response particularly in the 2F1+2F2, intermodulation, component. Since the major output of the second order neurons is in the medulla, this component arises there. It remains possible that some of the 2F1+2F2 component arises in more central parts of the visual system, but these are further from the recording site and so less likely to be involved.

References:

Afsari F, Christensen KV, Smith GP, Hentzer M, Nippe OM, Elliott CJ, and Wade AR (2014) Abnormal visual gain control in a Parkinson's disease model. Hum Mol Genet 23:4465-4478.

Gengs C, Leung HT, Skingsley DR, Iovchev MI, Yin Z, Semenov EP, Burg MG, Hardie RC and Pak WL (2002) The target of *Drosophila* photoreceptor synaptic transmission is a histamine-gated chloride channel encoded by ort (hclA). J Biol Chem 277:42113-42120.

Sweeney ST, Broadie K, Keane J, Niemann H, and O'Kane CJ (1995) Targeted expression of tetanus toxin light chain in *Drosophila* specifically eliminates synaptic transmission and causes behavioral defects. Neuron 14:341-351.

Supplementary Table 1. Statistical analysis of the SSVEP Contrast response function shown in Figure 2. Overall MANOVA followed by ANOVA of the three components in the SSVEP response

Overall	Pillai	F	P	
genotype	0.60489	18.3711	2.13E-07	***
timepoint	0.41412	8.482	0.0002155	***
genotype:timepoint	0.25708	4.1524	0.0126175	*

1F1 photoreceptor response

genotype		10.5611	0.0024205	**
timepoint		12.9101	0.0009248	***
genotype:timepoint		0.1414	0.7090138	NS

2F1 lamina neurons

genotype		0.5377	0.46789	NS
timepoint		19.335	8.55E-05	***
genotype:timepoint		4.7006	0.03648	*

2F1+2F2 medulla neurons

genotype		1.5958	0.214193	
timepoint		9.9119	0.003191	**
genotype:timepoint		0.5251	0.47313	
